# Αlpha 5 subunit-containing GABA_A_ receptors in temporal lobe epilepsy with normal MRI

**DOI:** 10.1093/braincomms/fcaa190

**Published:** 2021-01-07

**Authors:** Colm J McGinnity, Daniela A Riaño Barros, Rainer Hinz, James F Myers, Siti N Yaakub, Charlotte Thyssen, Rolf A Heckemann, Jane de Tisi, John S Duncan, Josemir W Sander, Anne Lingford-Hughes, Matthias J Koepp, Alexander Hammers

**Affiliations:** 1 Centre for Neuroscience, Department of Medicine, Imperial College London, London W12 0NN, UK; 2 MRC Clinical Sciences Centre, Hammersmith Hospital, London W12 0NN, UK; 3 King's College London & Guy's and St Thomas' PET Centre, School of Biomedical Engineering & Imaging Sciences, King’s College London, London SE1 7EH, UK; 4 Wolfson Molecular Imaging Centre, University of Manchester, Manchester M20 3LJ, UK; 5 Medical Image and Signal Processing (MEDISIP), Department of Electronics and Information Systems, Faculty of Engineering and Architecture, Ghent University, 9000 Ghent, Belgium; 6 Department of Medical Radiation Sciences, Institute of Clinical Sciences, Sahlgrenska Academy, University of Gothenburg, 413 45 Gothenburg, Sweden; 7 NIHR University College London Hospitals Biomedical Research Centre, UCL Queen Square Institute of Neurology, London WC1N 3BG, UK, and Chalfont Centre for Epilepsy, Chalfont St Peter SL9 0RJ, UK; 8 Stichting Epilepsie Instellingen Nederland (SEIN), Heemstede 2103SW, The Netherlands; 9 Neuropsychopharmacology Unit, Centre for Psychiatry, Department of Brain Sciences, Faculty of Medicine, Imperial College London, London W12 0NN, UK; 10 Neurodis Foundation, CERMEP, Imagerie du Vivant, 69003 Lyon, France

**Keywords:** GABA_A_, α5, memory, [^11^C]Ro15-4513, PET

## Abstract

GABA_A_ receptors containing the α5 subunit mediate tonic inhibition and are widely expressed in the limbic system. In animals, activation of α5-containing receptors impairs hippocampus-dependent memory. Temporal lobe epilepsy is associated with memory impairments related to neuron loss and other changes. The less selective PET ligand [^11^C]flumazenil has revealed reductions in GABA_A_ receptors. The hypothesis that α5 subunit receptor alterations are present in temporal lobe epilepsy and could contribute to impaired memory is untested. We compared α5 subunit availability between individuals with temporal lobe epilepsy and normal structural MRI (‘MRI-negative’) and healthy controls, and interrogated the relationship between α5 subunit availability and episodic memory performance, in a cross-sectional study. Twenty-three healthy male controls (median ± interquartile age 49 ± 13 years) and 11 individuals with MRI-negative temporal lobe epilepsy (seven males; 40 ± 8) had a 90-min PET scan after bolus injection of [^11^C]Ro15-4513, with arterial blood sampling and metabolite correction. All those with epilepsy and six controls completed the Adult Memory and Information Processing Battery on the scanning day. ‘Bandpass’ exponential spectral analyses were used to calculate volumes of distribution separately for the fast component [*V*_F_; dominated by signal from α1 (α2, α3)-containing receptors] and the slow component (*V*_S_; dominated by signal from α5-containing receptors). We made voxel-by-voxel comparisons between: the epilepsy and control groups; each individual case versus the controls. We obtained parametric maps of *V*_F_ and *V*_S_ measures from a single bolus injection of [^11^C]Ro15-4513. The epilepsy group had higher *V*_S_ in anterior medial and lateral aspects of the temporal lobes, the anterior cingulate gyri, the presumed area tempestas (piriform cortex) and the insulae, in addition to increases of ∼24% and ∼26% in the ipsilateral and contralateral hippocampal areas (*P* < 0.004). This was associated with reduced *V*_F_:*V*_S_ ratios within the same areas (*P* < 0.009). Comparisons of *V*_S_ for each individual with epilepsy versus controls did not consistently lateralize the epileptogenic lobe. Memory scores were significantly lower in the epilepsy group than in controls (mean ± standard deviation −0.4 ± 1.0 versus 0.7 ± 0.3; *P* = 0.02). In individuals with epilepsy, hippocampal *V*_S_ did not correlate with memory performance on the Adult Memory and Information Processing Battery. They had reduced *V*_F_ in the hippocampal area, which was significant ipsilaterally (*P* = 0.03), as expected from [^11^C]flumazenil studies. We found increased tonic inhibitory neurotransmission in our cohort of MRI-negative temporal lobe epilepsy who also had co-morbid memory impairments. Our findings are consistent with a subunit shift from α1/2/3 to α5 in MRI-negative temporal lobe epilepsy.

## Teaser

GABA_A_ receptors containing the α5 subunit (α5) mediate tonic inhibition and impair memory. McGinnity *et al.* imaged α5 and compared individuals with TLE, but normal structural imaging, and healthy controls. Patients had reduced memory performance and bilaterally higher temporal lobe α5, but lower α1/2/3 in the ipsilateral hippocampus.

## Graphical Abstract

**Graphical Abstract:**
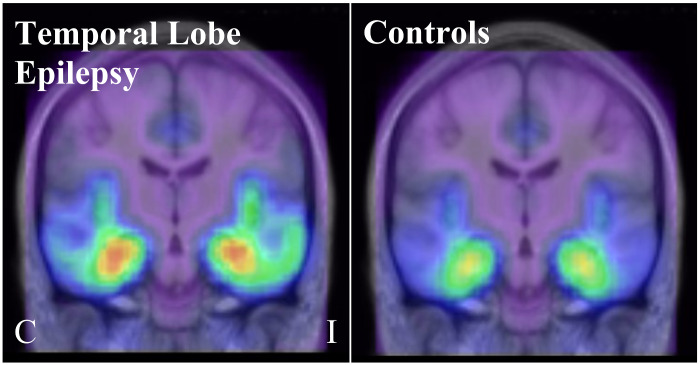

## Introduction

Gamma-aminobutyric acid (GABA) is the main inhibitory neurotransmitter in the brain (Curtis *et al.*, 1970) and mediates neurotransmission at 25–50% of central nervous system synapses. GABA_A_ receptors are ligand-gated chloride ion channels mediating phasic (synaptic) inhibitory neurotransmission and tonic (extrasynaptic) neurotransmission. The pentameric GABA_A_ receptor is assembled from 5 of 19 known protein subunit types α_1–6_, β_1–3_, δ, ε, γ_1–3_, π, ρ_1-3_ and θ (Barnard *et al.*, 1998). These receptor subunits exhibit distinct but overlapping distributions within the brain (Sieghart and Sperk, 2002), confer the pharmacological properties of the receptor, and have roles that change during development and with pathologies (Galanopoulou, 2008).

Approximately 5% of GABA_A_ receptors contain the α5 subunit (review: Jacob, 2019); they are predominantly, but not solely, extrasynaptic in localization (Brunig *et al.*, 2002; Brady and Jacob, 2015) and mediate tonic inhibitory currents (Caraiscos *et al.*, 2004).

Experiments in animals suggest that activation of receptors containing this subunit impairs hippocampus-dependent learning and memory: inverse agonism of the subunit has a positive effect on spatial learning (Chambers *et al.*, 2004; Sternfeld *et al.*, 2004; Dawson *et al.*, 2006) and antagonism enhances object recognition memory (Ling *et al.*, 2015; Gacsalyi *et al.*, 2017; 2018). Similar to the pharmacological studies, knock-out studies have shown that in α5 null mutant mice, associative learning (Yee *et al.*, 2004), spatial learning (Collinson *et al.*, 2002) and trace fear conditioning (Crestani *et al.*, 2002) are enhanced; conversely the *amnestic* effect of the anaesthetic etomidate on spatial and non-spatial learning is reduced (Cheng *et al.*, 2006). Data in humans are scarce; however, the amnestic effect of alcohol on word list learning is reduced by pre-treatment with an inverse α5 agonist (Nutt *et al.*, 2007). This suggests that α5 subunit availability is likely to adversely affect learning and memory, possibly by regulation of the threshold required for long-term potentiation (Martin *et al.*, 2010; Pofantis and Papatheodoropoulos, 2014).

The hippocampus and neighbouring medial temporal lobe structures are crucial to episodic memory function (Eichenbaum *et al.*, 2012). Focal epilepsies and in particular temporal lobe epilepsy (TLE) are strongly associated with verbal and visual memory disturbances, which were present in over half of a large cohort of individuals with focal epilepsy in Hoppe *et al.* (2007). In view of these learning and memory studies, one might hypothesize increased α5 subunit availability in individuals with TLE, either causally or as an epiphenomenon. Data on the expression of the α5 subunit in brain tissue resected from individuals with TLE is lacking. Alpha 5 subunit availability was, however, found to be *decreased* in the hippocampus proper (cornu ammonis CA1–CA4; Lorente de Nó, 1934) during the chronic state, via *in situ* hybridization and immunohistochemistry, in rodent chemoconvulsant models using kainic acid (Schwarzer *et al.*, 1997; Tsunashima *et al.*, 1997; Sperk *et al.*, 1998) or pilocarpine (Houser and Esclapez, 1996; Brooks-Kayal *et al.*, 1998; Fritschy *et al.*, 1999; Houser and Esclapez, 2003; Scimemi *et al.*, 2005; Bethmann *et al.*, 2008), as well as in amygdala kindling (Bethmann *et al.*, 2008) and hippocampal electrically induced status epilepticus models (Nishimura *et al.*, 2005). The decreases occur in association with neuronal loss and also in its absence (Rice *et al.*, 1996; Houser and Esclapez, 2003). Increased α5 subunit availability has been reported in the molecular and, less consistently, granule cell layers of the dentate gyrus in kainic acid (Schwarzer *et al.*, 1997; Bouilleret *et al.*, 2000) and pilocarpine models (Rice *et al.*, 1996; Fritschy *et al.*, 1999; Houser and Esclapez, 2003).

Previous human studies have used the PET radioligand [^11^C]flumazenil ([^11^C]FMZ, [^11^C]Ro15-1788), which is selective for the GABA_A_ receptor subunits α1–3 and α5 (Maziere *et al.*, 1984). [^11^C]FMZ total volume of distribution (*V*_T_) was reduced in the hippocampi and other temporal lobe regions of individuals with refractory TLE and normal MRI, i.e. without structural correlate of memory impairment (Henry *et al.*, 1993; Savic *et al.*, 1993; Savic and Thorell, 1996; Szelies *et al.*, 1996; Ryvlin *et al.*, 1998; Koepp *et al.*, 2000; Lamusuo *et al.*, 2000; Szelies *et al.*, 2000; Hammers *et al.*, 2002). [^11^C]FMZ binding was positively correlated with interictal interval (i.e. latency since last seizure; Bouvard *et al.*, 2005) and negatively correlated with seizure frequency (Savic *et al.*, 1996; Laufs *et al.*, 2011). In individuals who had paired [^11^C]FMZ scans 1 week apart, the binding was lowest for the scan that was associated with the shorter interictal interval (Bouvard *et al.*, 2005). Binding of this radioligand is, however, more indicative of the expression of α1 rather than α5 subunits given the former’s 10-fold higher relative concentration (Sieghart and Sperk, 2002).

The imidazobenzodiazepine Ro15-4513 (ethyl-8-azido-5,6-dihydro-5-methyl-6-oxo-4H-imidazo-1,4-benzodiazepine-3-carboxylate; F. Hoffmann-La Roche AG, Basel, Switzerland) behaves as a (competitive) α5 subunit inverse agonist (Bonetti *et al.*, 1988) at pharmacological doses; it can lower the threshold for seizures at high doses (Lister and Nutt, 1988). Receptors that express α5 subunits have 10–15 times higher affinity to Ro15-4513 than those that do not (Luddens *et al.*, 1994). Competition studies in the rat *in vivo* showed that radiolabelled Ro15-4513 uptake was reduced to non-specific levels only by drugs that have affinity for the α5 subtype (flunitrazepam, RY80, Ro15-4513, L655,708), but not by the α1 selective agonist zolpidem (Lingford-Hughes *et al.*, 2002). In healthy human volunteers, pre-scan blocking of α1 by administration of zolpidem in healthy human volunteers did not significantly decrease [^11^C]Ro15-4513 *V*_T_ (Myers *et al.*, 2012).

The hippocampus is the structure with the highest concentration of α5 subunit-containing receptors in the human brain (Sur *et al.*, 1998); expression is also high throughout the other parts of the limbic system. An *ex vivo* study with the α5 subunit-selective radioligand [^3^H]L-655,708 suggested the presence of α5 subunits in ∼28% of GABA_A_ receptors in the human hippocampus (Sur *et al.*, 1998); they appeared especially concentrated in CA 1 and 3 (Pirker *et al.*, 2000). [^11^C]Ro15-4513 PET offers a unique means of investigating, *in vivo*, GABA_A_ receptors that contain the α5 subunit in particular, unlike [^11^C]FMZ PET and [^123^I]iomazenil SPECT, which are mainly indicative of the distribution of α1 subtype. [^11^C]Ro15-4513 quantified with several methods including spectral analysis has excellent test–retest reliability (McGinnity *et al.*, 2017).

We used [^11^C]Ro15-4513 to test

1. whether there are changes in GABA_A_ α5 subunit availability in individuals with TLE but unremarkable MRI,
2. whether these changes lateralize TLE and
3. whether increased α5 subunit availability is inversely related to memory performance in tests known to involve the hippocampus.

## Materials and methods

### Participants

The primary inclusion criterion for the epilepsy group was TLE with normal MRI results. Eleven such individuals (median ± interquartile range age 40 ± 7.5 years, 7 males, 9 right-handed, interictal interval 6 ± 25 days, all taking anti-epileptic mediation) were included (Table 1); they had been recruited from outpatient epilepsy clinics at the National Hospital for Neurology and Neurosurgery (Queen Square, London) and the Chalfont Centre for Epilepsy (Chalfont St Peter). For this study, six healthy male controls were recruited via local advertising, of whom five completed test–retest [^11^C]Ro15-4513 PET (McGinnity *et al.*, 2017). Imaging data were available for another 17 healthy male controls from other studies (Stokes *et al.*, 2014) for a total of 23 subjects (49.0 ± 13.0 years). The sample size was decided based on preliminary analyses which predicted a power of 92% to detect a 10% difference in hippocampal total *V*_T_ with an *α* of 0.05 for one individual with TLE against 10 controls. We defined the minimum difference of interest as 10% based on our experience with other radiotracers, reasoning that lesser differences would not be ‘meaningful’ or indeed appreciable on visual analyses (which would be important if the radiotracer is to have clinical utility). We used the hippocampus as the region of interest in our sample size calculation because of its importance in TLE and in episodic memory, as described in the Introduction section.

The diagnosis of TLE was based on history and seizure semiology, as well as prolonged and repeated interictal and ictal electroencephalography recordings (where available). The semiology that we deemed suggestive of TLE included *déjà vu*, epigastric sensation, impairment of awareness, orofacial automatisms, manual automatisms and arm posturing. Ictal electroencephalography data were available for 10 of the 11 individuals who were subsequently enrolled in the study. Exclusion criteria for both groups were history of substance abuse, inability to provide informed consent, suffering any contraindication for undergoing PET or MRI, positive screening result for illicit drugs (see below for details), general practitioner’s (family doctor’s) advice against participation and pathological modified Allen’s test for patency of the ulnar artery (Allen, 1929). Additional exclusion criteria for controls were history of either psychiatric or systemic medical condition, or regular medication(s). Participants underwent a urine drug cassette test for 11-nor-Δ9-11-nor-Δ9-tetrahydrocannabinol, morphine, amphetamine, benzoylecgonine (the main metabolite of cocaine), methamphetamine and oxazepam (Monitect©; BMC, CA, USA) before PET scanning. One individual with TLE (RTLE4) tested positive for oxazepam as he had been prescribed clobazam 10 mg *nocte* four times a week, which had last been taken 3.5 days prior to the scan. The remaining participants had negative tests. Five of the healthy controls were scanned twice for test–retest analyses (McGinnity *et al.*, 2017; only the first scan was used for the analyses described in this study); all other participants were scanned only once.

### Ethical considerations

The London—Riverside Health Research Ethics Committee granted ethical approval for this cross-sectional PET study (08/H0706/30). The UK’s Administration of Radioactive Substances Advisory Committee granted permission for the tracer use. All participants provided written informed consent in accordance with the Declaration of Helsinki.

### Radiochemistry

[^11^C]Ro15-4513 was produced on site by Hammersmith Imanet as described previously (Myers *et al.*, 2012).

### Dynamic PET and memory score data acquisition

PET scans were acquired at Hammersmith Imanet on a Siemens/CTI ECAT EXACT HR+ 962 camera (Knoxville, TN, USA; Adam *et al.*, 1997; Brix *et al.*, 1997) in 3D mode, as described previously (McGinnity *et al.*, 2017). Each participant had a 10-min transmission scan for attenuation correction, followed by a 90-min dynamic emission scan consisting of 24 frames of increasing length. [^11^C]Ro15-4513 was injected as an intravenous bolus of median 450 MBq (interquartile range 45.1 MBq) 30 s after the dynamic emission scan start. The individuals with TLE were closely observed for evidence of seizures throughout the scan (none detected). Frames were reconstructed using Fourier rebinning (FORE; Defrise *et al.*, 1997) and 2D filtered backprojection (ramp filter, kernel 2.0 mm full-width at half-maximum) into 63 transaxial images.

### Neuropsychological assessment

All 11 individuals with TLE and six controls completed the Adult Memory and Information Processing Battery (AMIPB; Coughlan and Hollows, 1985), which consists of verbal and non-verbal subtests, on the day of scanning. The battery, which is used clinically in the epilepsy surgery programme at the National Hospital for Neurology and Neurosurgery (UK), has been shown to distinguish groups with left TLE, right TLE and healthy controls (Bonelli *et al.*, 2010). The assessments were performed in a quiet room by a clinician who had been trained in use of the AMIPB (authors A.H. and D.R.B.), in accordance with the procedure described in Baxendale (2010).

For the verbal list-learning task, each participant was read a list of 15 common words, some of which are semantically related and after each presentation they were asked to recall aloud as many words as possible. Immediately after the fifth trial, the participant was read a second list of 15 ‘distractor’ words, and again asked to recall aloud as many of these as possible. Immediately following this, the participant was then asked to recall aloud as many of the original 15 words (that were read five times) as possible, without further repetition (reading) by the clinician. We report as ‘List Learning’ the total number of words correctly recalled over the five trials, with a maximum score of 75 (5 × 15). We report as ‘List Recall’ the number of the original 15 words correctly recalled without repetition, out of a maximum of 15.

For the design learning task, each participant was asked to study an abstract line drawing consisting of nine distinct features presented on a grid, for 10 s. The participant was then asked to draw the same design on a blank grid. Similar to the verbal list-learning task, this procedure was repeated for further four trials. Immediately after the fifth trial, the participant was asked to study a new distractor design, and then asked to draw it on a blank grid. Immediately following this, the participant was then asked to draw the original design (that had been studied five times), without further repetition (presentation) by the clinician. We report as ‘Figure Learning’ the total number of distinct features correctly drawn over the five trials, with a maximum score of 45 (5 × 9). We report as ‘Figure Recall’ the number of the original distinct features correctly drawn without repetition, out of a maximum of 9.

### Input function derivation

Continuous and intermittent discrete blood samples were collected to allow the subsequent generation of arterial parent plasma input functions as described previously. In brief, during the first 15 min, blood radioactivity was continuously monitored in a bismuth germanate detection system (Ranicar *et al.*, 1991). Intermittent discrete (10 ml) samples were taken before the scan (baseline) and at 10 time points after the scan start. These discrete samples were used to quantify plasma and whole-blood radioactivity, as well as to quantify the parent fraction of the radioligand via high-performance liquid chromatography. The plasma-over-blood ratio model and the metabolite model were fitted and also applied to the whole blood measurements between 0 and 15 min. Continuous parent plasma input functions were then derived as described previously(Hinz *et al.*, 2007; Hammers *et al.*, 2007b; Riaño Barros *et al.*, 2014; McGinnity *et al.*, 2017).

### MRI data acquisition and analysis

All participants had 3D T1-weighted MRI scans with approximately millimetric isotropic voxel sizes for co-registration and region definition. There was no gross cerebral abnormality on any of the T1-weighted images. The hippocampal volumes were calculated for each participant using multi-atlas propagation with enhanced registration (Heckemann *et al.*, 2010).

### PET data quantification

The dynamic PET images were de-noised and corrected for frame-by-frame movement using wavelets (Turkheimer *et al.*, 1999) as described previously (Hammers *et al.*, 2007b; McGinnity *et al.*, 2017).

Summation images (also known as ‘add’ or ‘static’ images) were created for the realigned frames 1–24, using Modelling, Input functions and Compartmental Kinetics—Parametric Maps version 5.4 software (in-house MATLAB-based software available on request from the developer and co-author Rainer Hinz) for use as the reference image during co-registration of the T1-weighted MRI data.

Quantification of *V*_T_ was performed on a voxel-by-voxel basis using Modelling, Input functions and Compartmental Kinetics—Parametric Maps to generate parametric images with spectral analysis (Cunningham and Jones, 1993) modified as ‘bandpass’ spectral analysis (Myers *et al.*, 2017) to allow separation of distinct, subunit-tailored tracer kinetics. First, a binary mask of the brain was created by omitting the lowest 5% of signal from the corresponding summation images, to spatially constrain the subsequent spectral analyses to voxels with sufficient signal. The slow component volume of distribution [*V*_S_; mostly reflective of binding to α5 subunit-containing receptors (Myers *et al.*, 2017)] was calculated using a frequency interval of 0.00063–0.00137 s^−1^, whereas the fast component volume of distribution (*V*_F_; mostly reflective of binding to α1/α2/α3 subunit-containing receptors (Myers *et al.*, 2012)] was calculated using a frequency interval of 0.00137–0.1 s^−1^. We derived these boundaries empirically via examination of spectra derived from regional spectral analyses of the hippocampi (α5 rich) and occipital lobes and cerebella (α5 poor).

### Spatial image manipulation

The *V*_T_ images for each participant were co-registered to their corresponding T1-weighted MR image via the corresponding summation images using SPM12 (www.fil.ion.ucl.ac.uk/spm). To enable voxel-by-voxel analyses, images acquired from individuals with TLE in whom the epileptogenic zone was presumed to lie within the right temporal lobe (five individuals) were right-left flipped before spatial normalization, so the epileptogenic side appeared on the left side in all. To ensure like-for-like comparison and to exclude spurious differences arising from the flipping of individuals with right TLE, a similar proportion of controls (10 of 23) also needed to be right-left flipped. Therefore, random subsets of images acquired from ten controls were right-left flipped before spatial normalization (10 permutations). T1-weighted MR images were normalized to a symmetrical template (Didelot *et al.*, 2010) in Montreal Neurological Institute space using the deformation fields generated via Unified Segmentation (Ashburner and Friston, 2005) as implemented in SPM12. The same deformation fields were applied to the co-registered *V*_S_ and *V*_F_ images, which were subsequently smoothed (SPM12) using an isotropic Gaussian kernel of 12 mm full width at half maximum.

### Statistical analyses

#### Age, Adult Memory and Information Processing Battery scores and hippocampal volumes

We compared age and hippocampal volumes (ipsilateral and contralateral, separately) between the groups by Mann–Whitney U-tests. As there were significant negative correlations between the list learning and list recall AMIPB subtests and age (both *P* = 0.04), we compared the AMIPB scores by Mann–Whitney U-tests after regression of age. To allow subsequent interrogation of the relationship between AMIPB scores and *V*_S_, whilst keeping the number of analyses acceptably low, we summarized the age-regressed AMIPB scores as a single component by principal component analysis. We also compared this component between groups via an additional Mann–Whitney U-test. We used a significance threshold of *P* < 0.05.

#### *V*_S_ and *V*_F_ comparisons of groups (TLE versus controls); of each case versus 23 controls; and relationship with Adult Memory and Information Processing Battery scores

We compared *V*_S_ and separately *V*_F_ between the TLE and control groups on a voxel-by-voxel basis using the normalized, smoothed *V*_s_ and *V*_F_ images. We performed non-parametric two-sample pseudo-T tests (10 000 permutations) using the SnPM toolbox in SPM12 (Nichols and Holmes, 2002), with variance smoothing (8 mm full width at half maximum), and with a global total *V*_T_ (i.e. α1/2/3/5 subunits) covariate (Table 1). The images were (explicitly) masked at a relative threshold of 0.8. Ten tests were performed, corresponding to 10 control group flipping permutations. We chose to adopt the two-step ‘suprathreshold’ cluster test approach to significance testing as it is known to be more sensitive than the single threshold approach (Friston *et al.*, 1994; Nichols and Holmes, 2002). We report differences at a visualization pseudo-T threshold set arbitrarily to 2.5 followed by a conservative cluster threshold of *P* < 0.05 (family-wise error corrected). In a *post-hoc* analysis, we repeated the above comparison for flipping permutation 1, using threshold-free cluster enhancement as implemented in FSL version 5.0.9 (https://fsl.fmrib.ox.ac.uk/fsl/fslwiki) with the ‘randomise’ tool, version 2.9.

**Table 1:** Individuals with MRI-negative TLE and healthy controls—clinical and demographic details

ID	Age/sex/ handedness	Semiology	Interictal EEG ± PET ± EEG-fMRI	Ictal EEG	Consensus TLE lateralization	Onset/ duration (years)	Interictal interval (days)/seizure frequency (per month)	Antiepileptic drugs	Summary AMIPB score	Hippocampal volume Left/right (mm^3^)	Global total *V*_T_
LTLE1	39/M/L	FA aura (déjà vu); FIA with orofacial automatisms (swallowing); manual automatisms (scratching nose); anomia and neologism	L T (scalp)	Unclear (scalp)	Left	19/20	2/1.5	VAL, ZON	0.70	1672/1840	3.24
LTLE2	40/M/R	FA aura (déjà vu, epigastric); FIA with orofacial automatisms (lip smacking, facial grimacing); left and separately right bimanual automatisms (fidgeting); leg fidgeting; GM	Anterior T L > R (scalp); L T ([^18^F]FDG PET); EEG-fMRI bi-T	L T and separately R T (scalp)	Left	26/14	16/9	LEV, VAL	−2.02	2207/2472	2.98
LTLE3	40/M/R	FIA with left manual automatisms (fidgeting) and right arm posturing; speech arrest then dysarthria; facial grimacing, short-term memory deficit; GM	L T and R FT (scalp)	L T (scalp)	Left	18/22	2/15.5	PGB, VAL	−1.54	2262/2351	2.84
LTLE4	48/F/L	FA aura (‘sickness’); FIA with dysarthria and dysphasia; GM	L T and generalized (scalp); normal ([^11^C]flumazenil PET)	L T (scalp); widespread L TP (intracranial)	Left	17/31	115/0.2	CBZ, LEV	0.45	1348/1485	2.40
LTLE5	64/M/R	FIA with speech arrest, dysarthria, orofacial automatisms (swallowing), right arm movements, confusion	L mid-T (scalp)	L T (scalp)	Left	21/43	6/6.5	CBZ	−1.60	1817/2213	3.16
LTLE6	40/M/R	FIA with agrammatical speaking; manual automatisms; orofacial automatisms (lip smacking, tongue protrusion); GM	L T (scalp ); L T ([^18^F]FDG PET)	R T (scalp)	Left	37/3	30/1	CBZ, LEV	0.11	2059/2012	3.23
RTLE1	46/F/R	FA aura (déjà vu); FIA; GM	T R > L (scalp)	R (scalp)	Right	15/31	2/3	LTG, VAL	0.02	2270/2288	2.88
RTLE2	32/F/R	FA aura (déjà vu); FIA with orofacial automatisms (swallowing and lip smacking); bimanual automatisms	L > R mid-T, post-T, sphenoidal (scalp); R T ([^18^F]FDG PET)	R ant-T and sphenoidal (scalp)	Right	21/11	5/6.5	CBZ, VAL	0.29	2187/1998	3.36
RTLE3	42/M/R	FA aura (nausea); FIA with short-term memory deficits; GM	R T (scalp interictal)	−	Right	36/6	304/0.2	LEV	0.25	1926/2037	2.62
RTLE4	39/M/R	FA aura (epigastric); FIA with bimanual (L > R) automatisms (jaw rubbing); orofacial automatisms; versive head turn to left; tonic left arm posturing	R mid T (scalp); R T ([^18^F]FDG PET)	R T (scalp)	Right	27/12	1/4.5	CBZ, intermittent CLB, LAC, OXC, VAL	1.00	2010/2353	2.98
RTLE5	54/M/R	FIA with orofacial automatisms (lip smacking); left manual automatisms; right arm posturing; repetitive inappropriate words; shaking limbs or walking;	L T (scalp); normal ([^18^F]FDG PET)	R T and separately L T (scalp); L (scalp)	Right	22/32	24/2.5	PHB, VAL	−1.27	1838/1885	2.72
**Median ± iqr/total**	**40.0 ± 7.5/** **7 M, 4 F**	**2 FIA only;** **3 FA and FIA;** **2 FIA and GM;** **4 FIA, FA and GM**			**6 Left,** **5 Right**	**21.0 ± 6.5/** **20.0 ± 19.5**	**6.0 ± 25.0/** **3.0 ± 5.30**	**5 CBZ, 1 CLB, 1 LAC, 4 LEV, 1 LTG, 1 OXC, 1 PBG, 1 PHB, 7 VAL, 1 ZON**	**0.11 ±. 1.78**	**2010 ± 370/** **2037 ± 378**	**2.98 ± 0.42**
**Healthy controls**	**49 ± 13/** **23 M, 0 F**	**--**			**--**	**--**	**--**	**--**	**0.65 ± 0.85** (n = 6)	**1657 ± 481/** **1940 ± 484**	**2.86 ± 0.37**
***Mann– Whitney P***	***0.36***	−			**--**	−	−	−	***0.06***	***0.003/0.03***	***0.31***

AMIPB, Adult Memory and Information Processing Battery; CBZ, carbamazepine; CLB, clobazam, last taken ∼3.5 days prior to scan; EEG, electroencephalography; F, female; FIA, focal seizures with impaired awareness; FA, focal seizures with awareness; fMRI, functional MRI; FT, fronto-temporal; GM, generalized motor seizures; ID, identifying code; iqr, interquartile range; L, left; LAC, lacosamide; LEV, levetiracetam; LTG, lamotrigine; M, male; mm, millimetres; OXC, oxcarbazepine; PHB, phenobarbitone; PGB, pregabalin; R, right; T, Temporal; TP, temporoparietal; VAL, valproate; *V*_T_, volume of distribution

Non-flipped *V*_S_ were also compared for each individual participant with TLE to controls (i.e. 1 versus 23) by non-parametric two-sample pseudo-T tests as described above.

We interrogated the relationship between summary AMIPB scores (Shapiro–Wilk, *P* = 0.02) and hippocampal *V*_S_ (ipsilateral and contralateral averaged) for the individuals with TLE separately, and in combination with the controls as a single group, using Spearman’s rank correlation coefficient. In a *post-hoc* analysis, we also performed a voxel-by-voxel conjunction analysis using all four AMIPB subtests, with correction for multiple comparisons using a false discovery rate of 0.05 (see Supplementary Material).

#### Exploratory analysis of interictal interval

Based on a reported association between [^11^C]FMZ binding and the interval between last seizure and PET (Savic *et al.*, 1996; Bouvard *et al.*, 2005; Laufs *et al.*, 2011), we interrogated the relationship between interictal interval (transformed via natural logarithm) and global *V*_S_ using Pearson’s correlation coefficient.

#### *Post-hoc* analysis

We performed a *post-hoc* comparison of *V*_S_ between the TLE and control groups using flipping permutation one, as described above but after the application of a wavelet-based resolution recovery technique (iterative SFS-RR; (Shidahara *et al.*, 2012; McGinnity *et al.*, 2013; Silva-Rodríguez *et al.*, 2016; see Supplementary Material). The results did not differ substantially from those derived from the original comparison (presented below), in either directionality or significance.

### Data availability

Anonymized data are available from the corresponding author upon request.

## Results

All 34 participants completed the PET and MRI scans. Their medication usage at the time of scanning is summarised in Table 1.

### Between-group differences in baseline variables

#### Injectate

There was a small (∼8%) but significant difference in injected dose between individuals with TLE and controls, but injected dose was not correlated with global *V*_S_ (*P* = 0.27). Median occupancies were at tracer doses, i.e. below 2.5%, in individuals with TLE and controls. Controls had a median 0.9% point higher occupancy, but occupancy was not correlated with global *V*_S_ (*P* = 0.29).

**Table 2:** AMIPB scores for the individuals with MRI-negative TLE and healthy controls

	LTLE	RTLE	TLE—all	**Controls** (*n* = 6)	Mann–Whitney U, *P*; Cohen’s *d*
List Learning (maximum attainable = 75)	42.9 ± 9.6	46.3 ± 9.6	44.5 ± 9.3	57.3 ± 9.3	9.0, 0.02, 1.4
List Recall (maximum attainable = 15)	7.3 ± 2.8	9.6 ± 2.8	8.3 ± 2.9	12.1 ± 2.9	9.5, 0.02, 1.3
Figure Learning (maximum attainable = 45)	29.0 ± 12.7	30.9 ± 12.7	29.9 ± 12.2	38.9 ± 12.2	19.0, 0.16, 0.8
Figure Recall (maximum attainable = 9)	5.6 ± 2.7	8.5 ± 2.7	6.9 ± 2.9	8.5 ± 2.9	22.0, 0.27, 0.6
Summary AMIPB score	−0.7 ± 1.3	0.0 ± 0.6	−0.4 ± 1.0	0.7 ± 0.3	9.0, 0.02, 1.2

Scores are provided as estimated marginal means ± standard deviations (after regression of age), for the individual subtests. Higher values indicate better performance. Mann–Whitney U-tests were performed on the residuals (all individuals with epilepsy versus controls). The ‘Summary AMIPB’ score was generated via principal component analyses of the four age-regressed AMIPB subtest scores, see ‘Materials and Methods’.

#### Age and hippocampal volumes

There was no significant difference between the groups in age. The individuals with TLE had significantly larger hippocampi than controls (left *P* = 0.003, right *P* = 0.03) (Table 1).

#### AMIPB scores

Individuals with TLE had worse memory performance than controls, with significant differences evident overall and on the ‘List’ subtests (all *P* = 0.02; Table 2). Individuals with left TLE performed worse than those with right TLE on all subtests.

### Volumes of distribution

The global total *V*_T_ for RTLE4, who had taken intermittent clobazam ∼3.5 days before the scan, did not significantly differ from those of the remaining individuals with TLE (Table 1). When included, the global total *V*_T_ for RTLE4 was the median value for the group.

**Table 3:** Significant differences in [^11^C]Ro15-4513 *V*_S_ between individuals with MRI-negative TLE and healthy controls

			Cluster level		Voxel level			
			Volume (mm^3^)	*P* _FWE-corr_	Pseudo-T	*x*, *y*, *z*, mm	Region name	Proportion of SnPM analyses
**TLE > controls**	Pattern 1 (8/10 flips)	Cluster 1	83 492 ± 4 650	0.002 ± 0.001	5.21 ± 0.31	32 −17 −16	Contralateral hippocampus	8/8
			Secondary maxima:		4.51 ± 0.26	−32 −15 −16	Ipsilateral hippocampus/temporal horn	8/8
					4.56 ± 0.28	12 10 −02	Contralateral caudate	6/8
					4.29 ± 0.00	−08 25 −02	Corpus callosum near ipsilateral caudate	2/8
	Pattern 2 (2/10 flips)	Cluster 1	46 960 ± 3776	0.005 ± 0.001	5.00 ± 0.10	33 −18 −18	Contralateral hippocampus	2/2
			Secondary maxima:		4.27 ± 0.06	10 12 −02	Contralateral caudate	2/2
					4.38	−06 28 −02	Corpus callosum near ipsilateral caudate	1/2
					4.02	04 26 −02	Corpus callosum near contralateral caudate	1/2
		Cluster 2	36 840 ± 712	0.008 ± 0.001	4.77 ± 0.05	−32 −16 −14	Ipsilateral hippocampus/temporal horn	2/2
			Secondary maxima:		4.01 ± 0.19	−39 03 −36	Ipsilateral anterior medial temporal lobe	2/2
					3.99 ± 0.12	−60 −27 −16	Ipsilateral inferior/middle temporal gyrus / posterior temporal lobe	2/2
**TLE < controls**	–	–	–	–	–	–	–	–

mm(^3^), (cubic) millimetres; pFWE-corr, *P*-value corrected for multiple comparisons via family-wise error correction. Pattern 1 refers to a single cluster extending to both ipsilateral and contralateral temporal lobes; Pattern 2 describes a very similar pattern where ipsilateral and contralateral clusters are not connected. See text for details.

**Table 4:** [^11^C]Ro15-4513 *V*_T_s within hippocampal areas

	Hippocampal area *V*_S_		Hippocampal area *V*_F_		Hippocampal area *V*_F_: *V*_S_ ratios	
	Ipsilateral	Contralateral	Ipsilateral	Contralateral	Ipsilateral	Contralateral
TLE	6.21 ± 0.91	6.57 ± 0.86	1.35 ± 0.43	1.39 ± 0.41	0.23 ± 0.15	0.22 ± 0.14
Controls	5.02 ± 0.91	5.21 ± 0.85	1.72 ± 0.43	1.67 ± 0.40	0.38 ± 0.17	0.35 ± 0.14
Mann–Whitney U, *P*; Cohen’s *d*	45.0 0.003, 1.3	27.0, 0.0002, 1.6	66.0, 0.03, 0.8	81.0, 0.09, 0.7	52.0, 0.006, 0.9	54.0, 0.008, 0.9

The areas of *V*_S_ increase were isolated by the union between the SPM clusters and hippocampal regions of the Hammersmith maximum probability atlas (see text). *V*_S_ and *V*_F_ are summarized as estimated marginal means ± standard deviations after regression of global total *V*_T_; Mann–Whitney U-tests and calculations of Cohen’s *d* were performed on the residuals. *V*_F_: *V*_S_ ratios are expressed as means ± standard deviations.

**Figure 1:**
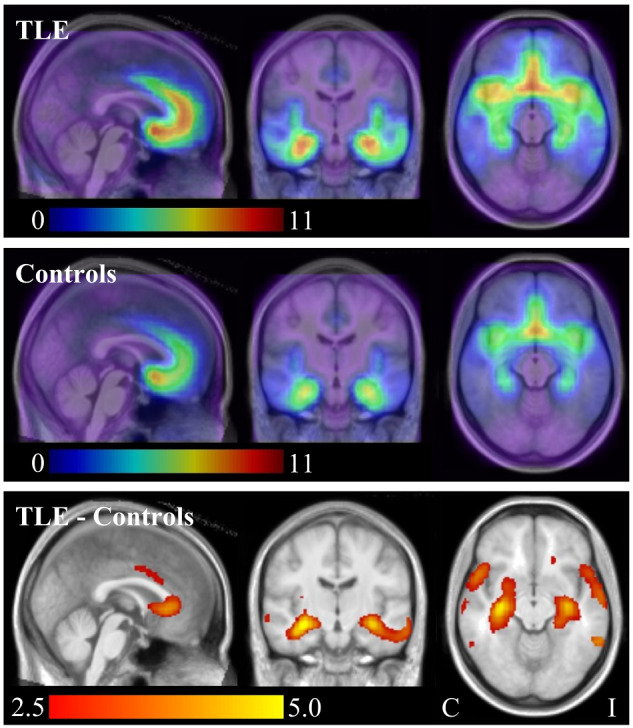
**Significant differences in [^11^C]Ro15-4513 *V*_S_ (individuals with MRI-negative TLE versus healthy controls).**  *Top row*—median for the TLE group (with flipping; ipsilateral is on the right of the image/left of the brain throughout); *middle row*—median for the control group; *Bottom row*—significant differences in *V*_S_ between the groups (red/yellow colour scale—TLE > Controls; cluster pseudo-T threshold 2.5). Bottom row depicts the median pseudo-T statistic image (10 random control flips). C, contralateral; I, ipsilateral

The TLE group had significantly higher *V*_S_ than the control group, with two similar patterns of clusters observed depending on random flipping. ‘Pattern 1’, which was returned for eight of the 10 random flips, consisted of one very large cluster with the peak voxel in the contralateral, anterior hippocampus in all eight random flips (83 492 ± 4650 mm^3^; cluster *P* = 0.002 ± 0.001 family-wise error corrected; Table 3; Fig. 1). This cluster also included a secondary peak in the ipsilateral, anterior hippocampus in all eight of these random flips. ‘Pattern 2’, which was returned for the remaining two random flips, consisted instead of two separate large clusters, one with peak voxel in the contralateral, anterior hippocampus for both flips (46  960 ± 3776 mm^3^, cluster *P* = 0.005 ± 0.001) and the other with peak in the ipsilateral hippocampus for both flips (46 960 ± 3776mm^3^, cluster *P* = 0.008 ± 0.001). Despite variation in the number of clusters, the increases in *V*_S_ were bilateral and extensive across all flips, and consistently encompassed anterior medial and lateral aspects of the temporal lobes, the anterior cingulate gyri, the presumed areas tempestas (piriform cortex) and the insulae (Fig. 1). The TLE group did not have any foci of significantly lower *V*_S_ than controls. Exclusion of the two individuals with TLE who had the longest interictal interval did not substantially alter these results.

Summary statistics for the ipsilateral and contralateral hippocampal areas of increase [isolated by the union between the clusters and hippocampal regions of the latest version of the Hammersmith maximum probability atlas (Faillenot *et al.*, 2017); https://brain-development.org/brain-atlases/adult-brain-atlases/] are provided in Table 4. In individuals with TLE, *V*_S_ was significantly higher than in controls in ipsilateral and contralateral hippocampal areas; *V*_F_ was significantly lower in the ipsilateral hippocampal area, and the *V*_F_:*V*_S_ ratio was significantly lower in ipsilateral and contralateral hippocampal areas.

**Figure 2:**
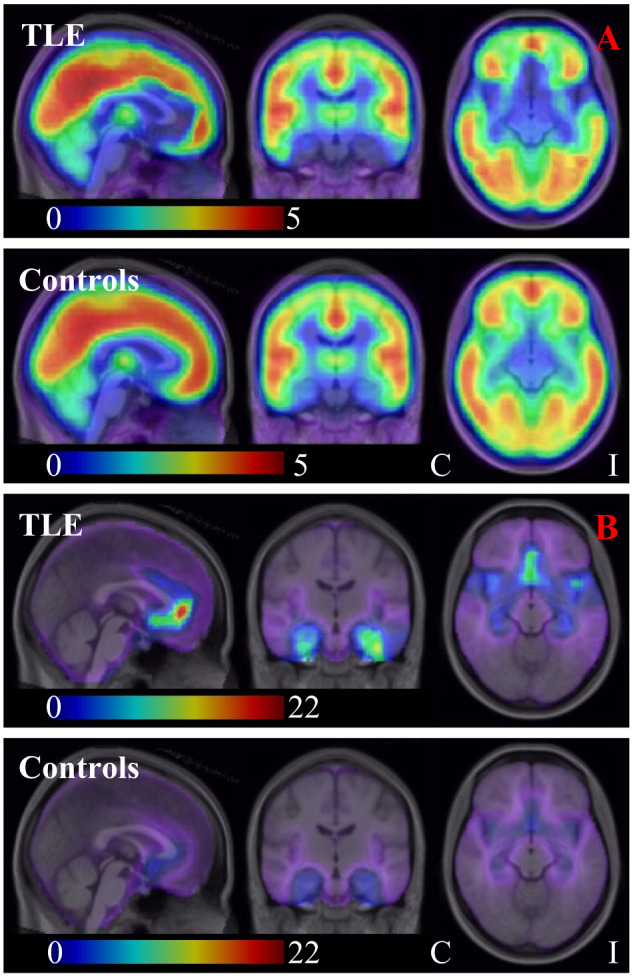
**[^11^C]Ro15-4513 *V*_F_ (**A**) and *V*_S_: *V*_F_ ratio (**B**; individuals with MRI-negative TLE versus healthy controls).** (**A**) median *V*_F_ for the TLE group (*upper row*; with flipping; ipsilateral is on the right of the image/left of the brain throughout) and for the control group (*lower row*); (**B**) median *V*_S_: *V*_F_ ratio for the TLE group (*upper row*; with flipping; ipsilateral is on the right of the image/left of the brain throughout) and for the control group (*lower row*). Note that in **B**, for illustrative purposes, we depict *V*_S_: *V*_F_ ratio rather than *V*_F_: *V*_S_ ratio. C, contralateral; I, ipsilateral

There were no significant differences in *V*_F_ between the TLE group and the control group (Fig. 2).

The *post-hoc* analysis for flipping permutation 1 using threshold-free cluster enhancement yielded results that were virtually identical to those generated using the two-step ‘suprathreshold’ cluster test approach (see Supplementary Fig. 3).

### Comparisons of *V*_S_ in each case versus 23 controls

Two controls had areas of *V*_S_ that significantly differed from that of the remaining controls (one control with an increase, one control with a decrease; i.e. two of 46 comparisons, 4.3%; cluster *P* < 0.05 family-wise error corrected). At the same thresholds, two of the individuals with TLE had a significant increase each, one of which was localized to the ipsilateral frontal and temporal lobes and the other to ipsi- and contralateral frontal and temporal lobes (but with an ipsilateral frontal peak; see Supplementary Figs 1 and 2). There were no significant decreases in *V*_S_.

### *V*_S_ and AMIPB scores

**Figure 3:**
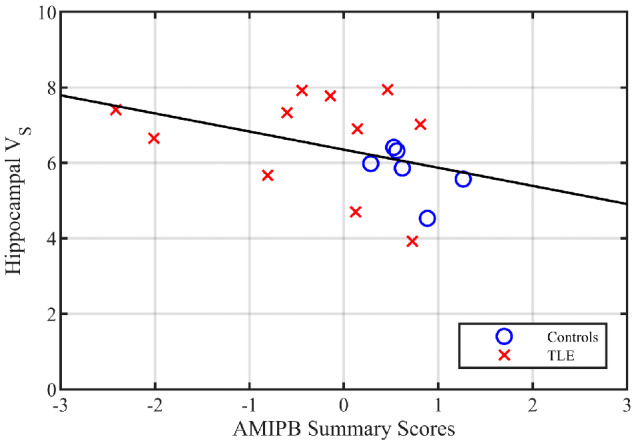
**Hippocampal *V*_S_ versus summary AMIPB scores.** AMIPB—Adult Memory And Information Processing Battery. The *V*_S_ (i.e. slow component volume of distribution) was summarized as the mean of the left and right hippocampi. The age-regressed AMIPB scores were summarized as a single component by principal component analyses. Higher AMIPB summary scores indicate better memory performance

There were no significant correlations between the summary AMIPB scores and mean hippocampal *V*_S_ (individuals with TLE *ρ* = −0.09, *P* = 0.79; controls and individuals with TLE combined as a single group *ρ* = −0.48, *P* = 0.05). The negative associations between summary AMIPB score and hippocampal *V*_S_ are illustrated in Fig. 3.

The *post-hoc* voxel-by-voxel analysis did not yield any clusters of conjunction of significance.

### Exploratory analysis of *V*_S_ and interictal interval

**Figure 4:**
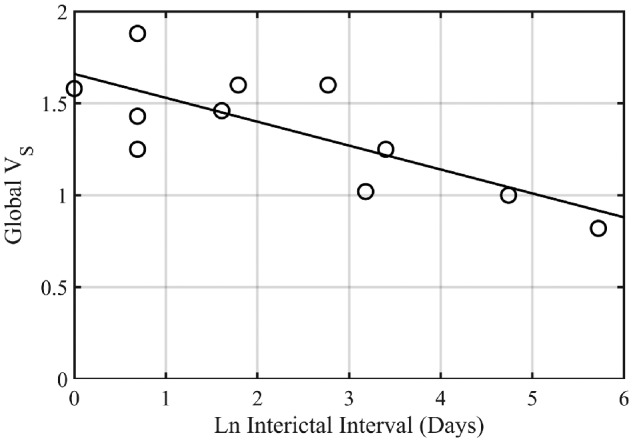
**Global *V*_S_ versus interictal interval.** The interictal interval has been transformed via the natural logarithm (Ln). Pearson’s correlation coefficient (*r*) was −0.77 (*P* = 0.005). V_S_, slow component volume of distribution.

The analysis revealed negative correlations between interictal interval transformed by natural logarithm and global *V*_S_ (*r* = −0.77, *P* = 0.005, Fig. 4). Additional *post-hoc* tests showed a significant negative correlation with the mean hippocampal area *V*_S_ (ipsilateral and contralateral averaged; *r* = −0.65, *P* = 0.03), but not with mean hippocampal area *V*_F_ or mean hippocampal area *V*_F_:*V*_T_ ratio (*r* = 0.32, *P* = 0.34; and *r* = 0.52, *P* = 0.10).

## Discussion

We used bandpass spectral analysis to reveal, for the first time in humans, several alterations of *α* subunit binding in MRI-negative TLE, including higher *V*_S_, i.e. mostly α5 hippocampal binding of [^11^C]Ro15-4513.

### Strengths and limitations

Our study’s principal strength is its robust PET methodology. [^11^C]Ro15-4513 has approximately nanomolar affinity for GABA_A_ receptors, with approximately 10–15 times higher affinity for those receptors containing α5 than for those that do not (Luddens *et al.*, 1994). We obtained metabolite-corrected arterial plasma input functions, the gold-standard approach for quantitative PET and report the first use of voxel-by-voxel bandpass spectral analysis, going beyond previous region-of-interest-based approaches (Mendez *et al.*, 2013; Stokes *et al.*, 2013; 2014; Horder *et al.*, 2018). Bandpass spectral analysis allows approximate separation of slow (*V*_S_; mostly α5) from other binding on the basis of cerebral tissue kinetics. This approach has permitted parametric imaging of two targets using [^11^C]Ro15-4513, and could conceivably be applied to other radioligands with selectivity for more than one target.

An additional strength is that we deliberately excluded individuals with visually apparent MRI abnormality, such as hippocampal sclerosis, to minimize the confounding influence of structural changes which might explain memory difficulties, and of partial volume effect. This may have biased our sample towards patients with larger-than-usual hippocampi, and we unexpectedly found that individuals with TLE in our sample had slightly larger hippocampi than controls (Table 1). However, in the *post-hoc* analysis, the application of a wavelet-based resolution recovery technique had no effect on the directionality or significance of the difference in *V*_S_ between the TLE and control groups. The difference between groups is also readily apparent even on visual inspection of the unfiltered images in native space. Therefore, we are confident that our findings are not driven by the partial volume effect.

Six of our individuals with TLE have had [^18^F]fluorodeoxyglucose or [^11^C]FMZ PET, which was localizing in four. Only one of our individuals with TLE has had intracranial electroencephalography; therefore mis-localization in some cases is possible. This limits the inference that can be made on the lateralizing capacity of [^11^C]Ro15-4513 *V*_S_, but does not invalidate the association we have observed between refractory focal epilepsy and increased α5 subunit expression.

Four of our individuals with TLE were female, whereas all of our controls were male; we are unaware of any studies on the effect of gender or the menstrual cycle on α5 subunit expression, but cannot exclude this as a potential confound.

High-dose valproic acid was associated with an unexpected doubling of hippocampal and amygdala [^11^C]Ro15-4513 standardized (α1/2/3/5) uptake in rats, using standardized uptake values (α1/2/3/5) without an arterial input function to correct for possible differences in metabolism or blood flow (Bertelsen *et al.*, 2018). We checked whether the individuals with TLE who were taking sodium valproate at the time of the scan in our study had different *V*_S_, *V*_F_ and *V*_F_:*V*_S_ ratios in the hippocampal areas compared to the other individuals, but did not find a significant difference (*P* ≥ 0.19).

### α5 subunit expression and seizures

As noted in the Introduction, increases and decreases in α5 subunit expression have been reported in animal models. The reason for discrepant findings in the preclinical literature is not clear, however, differences in experimental procedure such as in the timing of sacrifice and the region selected for examination might be important. Our finding of substantial increases (∼25%) in hippocampal limbic lobe *V*_S_ in human TLE is consistent with the reports of increased dentate gyrus α5 subunit expression in models using kainic acid (Schwarzer *et al.*, 1997; Bouilleret *et al.*, 2000) and pilocarpine (Rice *et al.*, 1996; Fritschy *et al.*, 1999; Houser and Esclapez, 2003). Notably, the increases we describe extend well beyond the hippocampus into other regions of the lateral temporal lobe, which have not been scrutinized in animal models. Our findings highlight the need for validation of data derived from experimental models in humans, *in vivo*.

The finding of higher limbic lobe *V*_S_ in those with refractory TLE suggests either: (i) upregulation of α5 subunit expression occurring in as a consequence of spontaneous epileptic seizures; or (ii) chronic upregulation of α5 subunit expression resulting in a vulnerability to seizures. Longitudinal study would allow these hypotheses to be formally tested. From our data, the finding of a negative correlation of *V*_S_ with interictal interval favours the first explanation. We interpret the increase in α5 binding we observed in individuals with TLE as an increase in receptor concentration, but it could also reflect slowing of the cerebral tissue kinetics.

Upregulation of expression during the early interictal phase would result in increased tonic inhibitory neurotransmission, which we suggest may contribute to suppression of further seizures (Sackeim *et al.*, 1987). Our findings complement earlier reports of dynamic alterations in cannabinoid (Goffin *et al.*, 2011), GABA_A_ (Savic *et al.*, 1996; Bouvard *et al.*, 2005) and opioid receptor binding in association with temporal lobe seizures (Hammers *et al.*, 2007*a*; McGinnity *et al.*, 2013).

Receptor upregulation following seizures is not invariable; it should be noted, for example, that hippocampal [^11^C]FMZ binding, reflective of availability of α subunits 1–3 and 5, was actually lower in individuals with MRI-negative TLE (Koepp *et al.*, 2000; Hammers *et al.*, 2002) and in individuals with TLE with hippocampal sclerosis (Koepp *et al.*, 1996; 1997; Hammers *et al.*, 2001). In six individuals with TLE (three of whom were MRI-negative) who had paired [^11^C]FMZ scans one week apart, the binding was lowest for the scan that was associated with the shorter interictal interval (Bouvard *et al.*, 2005), and the extent of reduction in [^11^C]FMZ binding in the PET abnormality (relative to the contralateral homologue) was positively correlated with seizure frequency in 17 individuals with MRI-negative focal (mostly temporal lobe) epilepsy (Savic *et al.*, 1996). [^11^C]FMZ binding in the presumed area tempestas, near the piriform cortex, was also negatively correlated with seizure frequency in 18 individuals with MRI-negative focal (mostly frontal) lobe epilepsy (Laufs *et al.*, 2011).

Together, therefore, these data suggest that a ‘subunit shift’ may effectively occur in TLE, from α subunits 1/2/3 to 5, in response to seizures. The results of our exploratory and *post-hoc* analyses of the associations between global and, separately, hippocampal area *V*_S_ and interictal interval support this hypothesis, and were complemented by the identification of significantly lower *V*_F_s and *V*_F_:*V*_S_ ratios for the individuals with TLE than for controls in the same hippocampal areas. Such a shift might be expected to predispose the individual to learning and memory impairments, consistent with the TLE clinical phenotype (Giovagnoli and Avanzini, 1999).

[^11^C]Ro15-4513 *V*_S_ does not appear to lateralize MRI-negative TLE, which, while disappointing in terms of usefulness for pre-surgical evaluation, is consistent with asymmetric but to a degree bilateral decreases in [^11^C]FMZ binding (Ryvlin *et al.*, 1998; Hammers *et al.*, 2002). Caution is required in the interpretation of this result due to potential mis-localization.

### α5 subunit expression and memory impairment

Our finding of substantial increases (∼25%) in hippocampal limbic lobe *V*_S_ in human TLE is also consistent with co-morbid memory impairments in individuals with TLE, given the extensive literature summarized in the Introduction which suggests activation of receptors containing the α5 subunit impairs hippocampus-dependent learning and memory. Contrary to our expectations and findings in individuals with alcohol dependence (Lingford-Hughes *et al.*, 2012), however, we did not detect any significant correlation between summary AMIPB scores and hippocampal *V*_S_ for the individuals with TLE (but observed a borderline significant correlation when pooled with the controls). Caution is required in the interpretation of this result due to the sample size, pooling of individuals with left and right TLE, and potential mis-localization. Whilst the performance of our individuals with TLE on the List Learning and Figure Learning AMIPB subtests was similar to those reported in a larger study (Bonelli *et al.*, 2010), our ability to detect impairments was compromised by a ceiling effect observed in the non-verbal (figure) recall tests, where several individuals were able to attain the maximum possible score. The use of a delayed recall task might have better indexed hippocampal-dependent memory than the AMIPB subtests, which quantify immediate/short-term recall.

## Conclusion

Our results provide evidence for increased tonic inhibitory neurotransmission in MRI-negative TLE. The increases in *V*_S_ were bilateral and therefore not useful for lateralization of TLE. While causal inferences cannot be made, the finding of increased [^11^C]Ro15-4513 *V*_S_ is consistent with the co-morbid memory impairments in this population. Exploratory analyses suggested a relationship with time since last seizure.

## Supplementary material

Supplementary material is available at *Brain Communications* online.

## Supplementary Material

fcaa190_Supplementary_Data

## Abbreviations

[^11^C]FMZ =: [^11^C]flumazenil
AMIPB =: Adult Memory and Information Processing Battery
CA =: cornu ammonis
GABA =: gamma-aminobutyric acid
LTLE =: left temporal lobe epilepsy
MR =: magnetic resonance
TLE =: temporal lobe epileps
y
*V*_F_ =: fast component volume-of-distribution
*V*_S_ =: slow component volume-of-distribution
*V*_T_ =: (total) volume-of-distribution
