# Supplementary material for: Αlpha 5 subunit-containing GABAA receptors in temporal lobe epilepsy with normal MRI
Source: Brain Commun. 2021 Jan 7;3(1):fcaa190. doi: 10.1093/braincomms/fcaa190 (PMC7811756; doi:10.1093/braincomms/fcaa190)
Supplement: fcaa190_Supplementary_Data [file fcaa190_supplementary_data.docx]

Αlpha 5 subunit-containing GABA_A_ receptors in temporal lobe epilepsy with normal MRI: Supplementary Material

## Comparisons of V_S_ in each case versus 23 controls


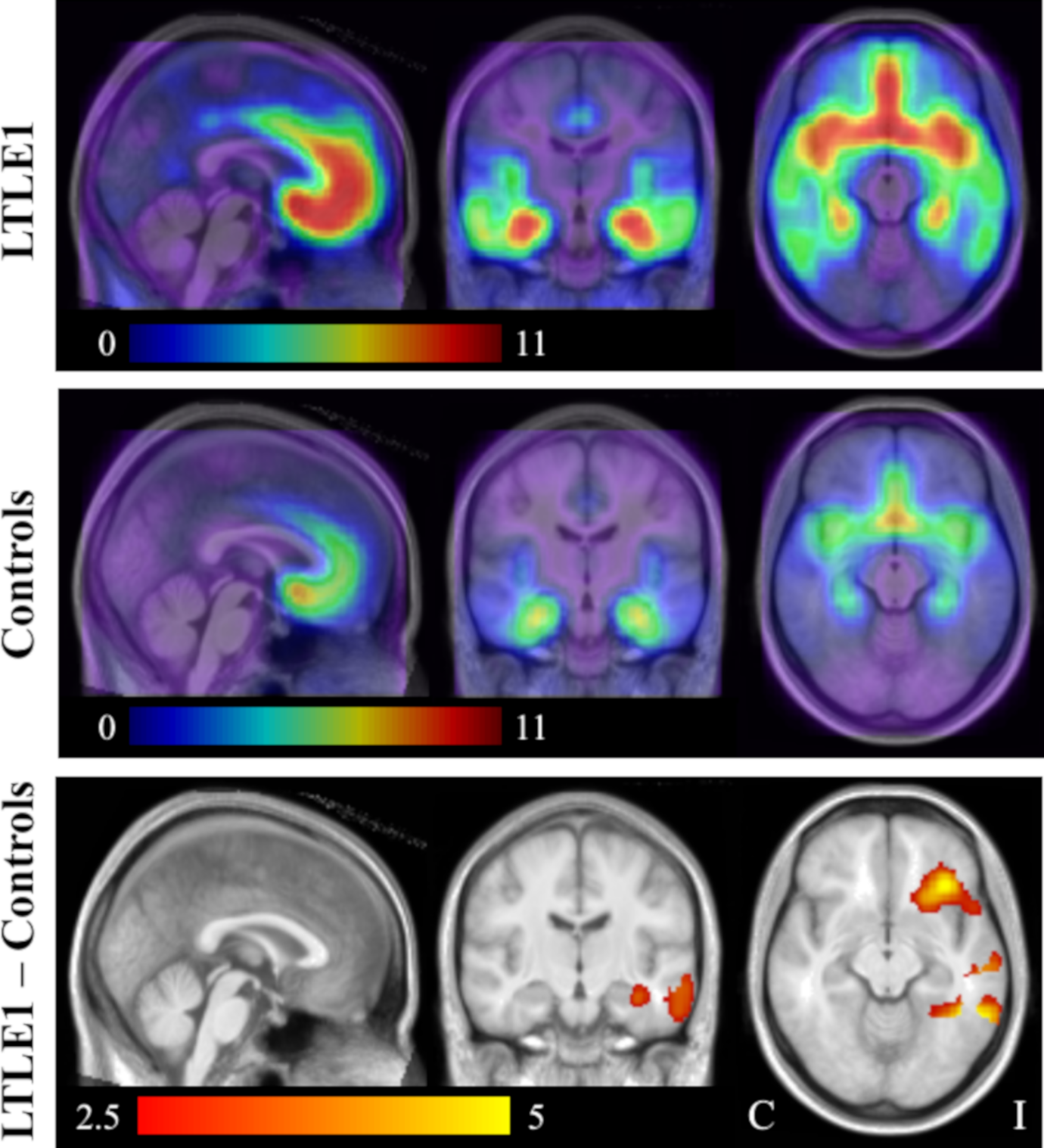


**Supplementary Fig. 1: Significant differences in [^11^C]Ro15-4513 V_S_ (LTLE1 versus healthy controls).** Top pane – LTLE1 (with flipping; ipsilateral is on the right of the image / left of the brain throughout). Middle pane – median for the control group; bottom pane shows significant differences in V_S_ between the LTLE1 and the control group (red/yellow colour scale – TLE>Controls; cluster pseudo-T threshold 2.5). Note that distribution of significant clusters appears more bilateral than depicted here when a less stringent statistical threshold is used (e.g. p < 0.05 uncorrected rather than p < 0.05 with family-wise error correction). C – contralateral; I – ipsilateral.


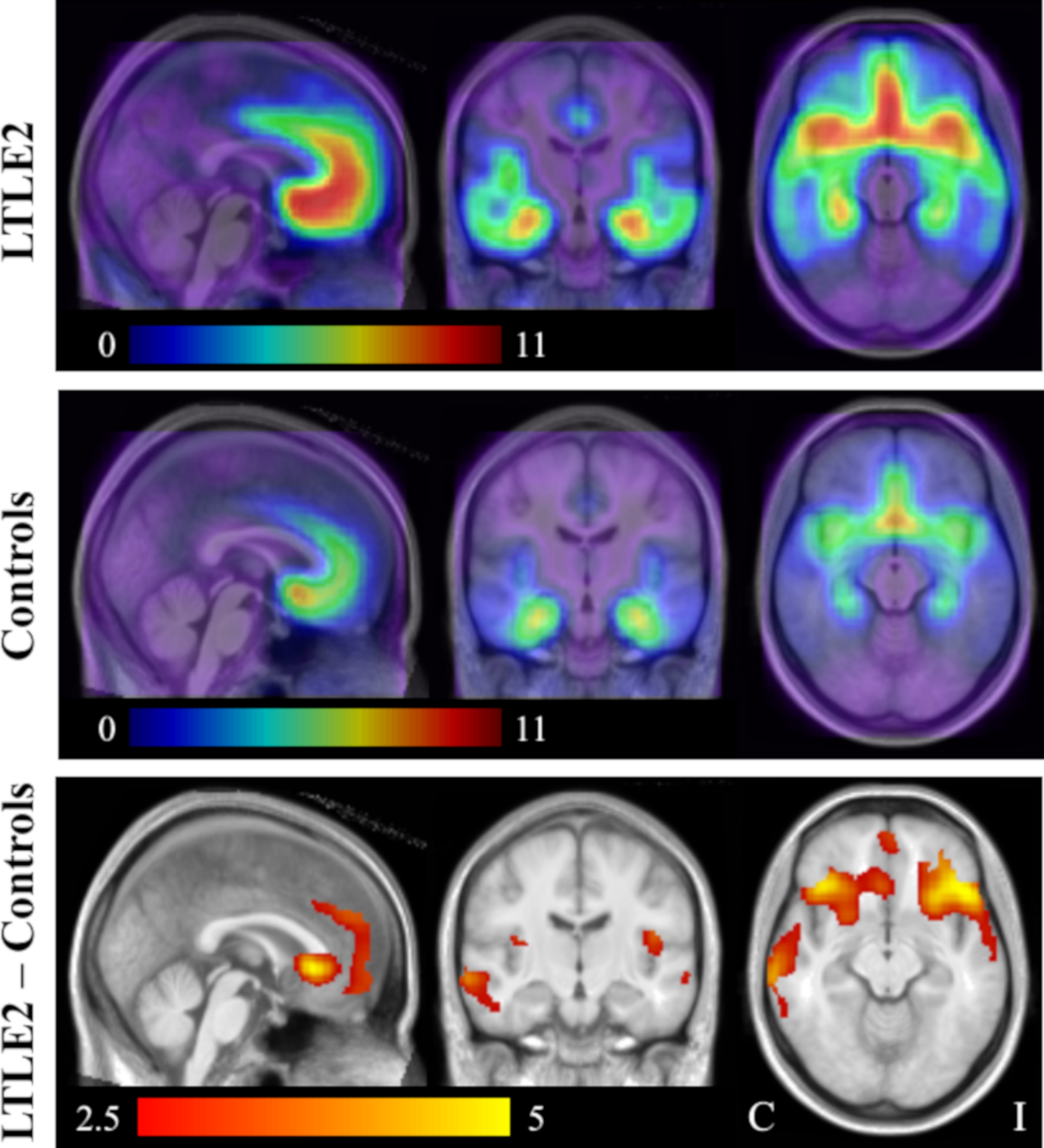


**Supplementary Fig. 2: Significant differences in [^11^C]Ro15-4513 V_S_ (LTLE2 versus healthy controls).** Top pane – LTLE2 (with flipping; ipsilateral is on the right of the image / left of the brain throughout). Middle pane – median for the control group; bottom pane shows significant differences in V_S_ between the LTLE1 and the control group (red/yellow colour scale – TLE>Controls; cluster pseudo-T threshold 2.5). C – contralateral; I – ipsilateral.

## Application of Structural – Functional Synergistic Resolution Recovery (SFS-RR)

In a post-hoc analysis, we applied iterative SFS-RR ((Shidahara *et al.*, 2012; McGinnity *et al.*, 2013; Silva-Rodríguez *et al.*, 2016); 6 iterations) to the total volume-of-distribution (V_T_) images using individualised atlases that were derived from the application of MAPER the participant’s corresponding T1-weighted MR image (Heckemann *et al.*, 2006; Heckemann *et al.*, 2010). We used the V_T_ images was used rather than V_S_ images because they had a higher signal-to-noise ratio. We generated a scaling factor image for each participant by dividing the output, resolution-recovered image by the uncorrected V_T_ image. The uncorrected V_S_ image for each participant was then multiplied by the corresponding scaling factor image to produce an output V_S_ image that was “corrected” for the partial volume effect. We compared the corrected V_S_ in between individuals with TLE and controls on a voxel-by-voxel basis, as described in the manuscript proper for the analysis, using flipping permutation one.

## Relationship with AMIPB scores and V_S_: conjunction analysis

In a post-hoc analysis, we interrogated the relationship between AMIPB subtest scores and Vs on a voxel-by-voxel basis by conjunction analysis, using the normalised, smoothed V_s_ images. We first performed non-parametric simple regression (10,000 permutations) for each of the four subtest scores, separately, using the SnPM toolbox in SPM12 (Nichols *et al.*, 2002). We used variance smoothing (8 mm FWHM), and a global total V_T_ (i.e. α1/2/3/5 subunits) covariate (Table 1). As there were significant negative correlations between the list learning and list recall AMIPB subtests and age (both p=0.04), age was entered as an additional covariate (Table 1). The images were (explicitly) masked at a relative threshold of 0.8.

Each simple regression yielded an image, “IP-.img”, of uncorrected –log_10_ p-values for the negative association. These were rescaled to p values by multiplying the image by minus one and then raising 10 to the power of the output (i.e. 10^^-i1^; where i1 is the “IP-.img”). We took the maximum of these four p-value images and submitted to false discovery rate correction at 0.05 using the Matlab function provided by the NeuroImaging Statistics Oxford (NISOx) research group (www.nisox.org/Software/fdr/).

## Volumes-of-distribution: threshold-free cluster enhancement


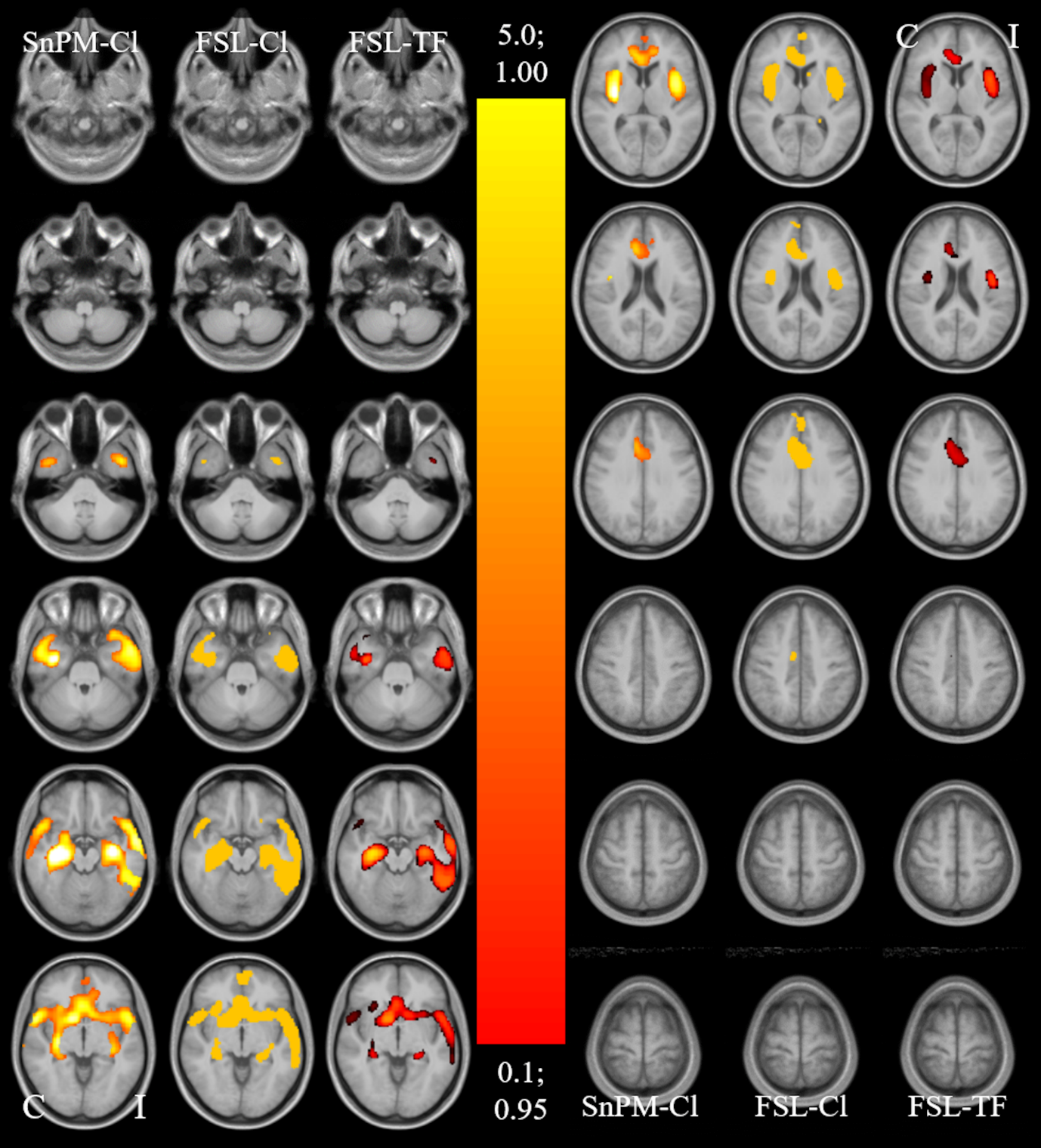


**Supplementary Fig. 3: Significant differences in [^11^C]Ro15-4513 V_S_ (TLE versus healthy controls).** SnPM-Cl – SnPM with the two-step “suprathreshold” cluster test approach; FSL-Cl – FSL with the two-step “suprathreshold” cluster test approach; FSL-TF – FSL with threshold-free cluster enhancement. SnPM images colour scale 0.1 – 5.0 (pseudo-T); FSL images colour scale 0.95 – 1 (1 minus p i.e. p of 0.05 – 0). C – contralateral; I – ipsilateral.

The distribution of significant clusters was virtually identical across the approaches (SnPM13 with cluster extent threshold 2.5; first and fourth columns), randomise 2.9 with cluster extent threshold 2.5 (second and fifth columns), and randomise 2.9 with TFCE (third and sixth columns).

Some minor variation in results is expected across software due to the specifics of the implementation; for example, relative threshold masking at 0.8 was used for the SnPM method but not for the randomise methods.

## References

Heckemann RA, Hajnal JV, Aljabar P, Rueckert D, Hammers A. Automatic anatomical brain MRI segmentation combining label propagation and decision fusion. Neuroimage 2006; 33: 115-26.

Heckemann RA, Keihaninejad S, Aljabar P, Rueckert D, Hajnal JV, Hammers A. Improving intersubject image registration using tissue-class information benefits robustness and accuracy of multi-atlas based anatomical segmentation. Neuroimage 2010; 51: 221-7.

McGinnity CJ, Shidahara M, Feldmann M, Keihaninejad S, Riano Barros DA, Gousias IS*, et al.* Quantification of opioid receptor availability following spontaneous epileptic seizures: correction of [^11^C]diprenorphine PET data for the partial-volume effect. Neuroimage 2013; 79: 72-80.

Nichols TE, Holmes AP. Nonparametric permutation tests for functional neuroimaging: a primer with examples. Hum Brain Mapp 2002; 15: 1-25.

Shidahara M, Tsoumpas C, McGinnity CJ, Kato T, Tamura H, Hammers A*, et al.* Wavelet-based resolution recovery using an anatomical prior provides quantitative recovery for human population phantom PET [^11^C]raclopride data. Phys Med Biol 2012; 57: 3107-22.

Silva-Rodríguez J, Cortés J, Rodríguez-Osorio X, López-Urdaneta J, Pardo-Montero J, Aguiar P*, et al.* Iterative Structural and Functional Synergistic Resolution Recovery (iSFS-RR) Applied to PET-MR Images in Epilepsy. IEEE Trans Nucl Sci 2016; 63: 2434-42.
